# Microparticles in aneurismal subarachnoid hemorrhage: role in acute and delayed cerebral ischemia

**DOI:** 10.1186/cc12278

**Published:** 2013-03-19

**Authors:** V Conte, A Artoni, S Magnoni, M Leoni, L Capuano, V Civelli, N Stocchetti

**Affiliations:** 1 Fondazione IRCCS Ca' Granda-Ospedale Maggiore Policlinico, Milan, Italy; 2 Fondazione IRCCS Ca' Granda-Ospedale Maggiore Policlinico, Milan University, Milan, Italy

## Introduction

Microthrombosis has been demonstrated in early and delayed cerebral ischemia after aneurysmal subarachnoid hemorrhage (aSAH). Markers of coagulation activation as microparticles (MPs) are an established risk factor for thrombosis. Our hypothesis was that levels of microparticles might correlate with aSAH severity, early cerebral ischemia (ECI) and delayed cerebral ischemia (DCI).

## Methods

Consecutive aSAH patients (age 18 to 80) admitted to our department between November 2011 and September 2012 were enrolled in the study. Total MPs (anxV+), platelet MPs (PLT-MPs, anx+/ CD41+), tissue factor MPs (TF-MPs, anx+/CD142+), and endothelial MPs (E-MPs, anx+/CD144+) were assessed in venous blood samples. Measurement of MPs levels were obtained at early (<3 days) and delayed phase (7 to 10 days). Multiparameter MRI (T2; FLAIR; T1; AngioRM 3D TOF HR; DWI) was performed to evaluate ischemic damage and vasospasm at the same time points. Qualitative evaluation of severity of ischemic damage was performed on DWI sequences. Levels of MPs were evaluated comparing different groups in terms of SAH severity (World Federation of Neurological Surgeons score or WFNS), ECI severity and occurrence of delayed complications (vasospasm and DCI).

## Results

Twenty-five patients (age 57 ± 12 years, WFNS 1 to 5) were included in the analysis. Overall increased levels of MPs were observed at early phase after aSAH when compared with controls. Contrarily to our hypothesis, in the early phase no significant differences of MP level were observed in WFNS 4 to 5 (*n = *9) when compared with WFNS 1 to 2 (*n = *16) patients. Interestingly, patients with most severe ECI and DCI showed the highest values of MPs, but this trend did not reach statistical significance, probably because of the small number of patients included in the study.

## Conclusion

Microparticles and microthrombosis may increase the severity of early and delayed ischemic damage after aSAH.

